# Nodal rings and drumhead surface states in phononic crystals

**DOI:** 10.1038/s41467-019-09820-8

**Published:** 2019-04-16

**Authors:** Weiyin Deng, Jiuyang Lu, Feng Li, Xueqin Huang, Mou Yan, Jiahong Ma, Zhengyou Liu

**Affiliations:** 1 School of Physics and Optoelectronics, South China University of Technology, 510640 Guangzhou, Guangdong China; 2 Key Laboratory of Artificial Micro- and Nanostructures of Ministry of Education and School of Physics and Technology, Wuhan University, 430072 Wuhan, China; 3 Institute for Advanced Studies, Wuhan University, 430072 Wuhan, China

## Abstract

Three-dimensional topological nodal lines, the touching curves of two bands in momentum space, which give rise to drumhead surface states, provide an opportunity to explore a variety of exotic phenomena. However, solid evidence for a flat drumhead surface state remains elusive. In this paper, we report a realization of three-dimensional nodal line dispersions and drumhead surface states in phononic crystal. Profiting from its macroscopic nature, the phononic crystal permits a flexible and accurate fabrication for materials with ring-like nodal lines and drumhead surface states. Phononic nodal rings of the lowest two bands and, more importantly, topological drumhead surface states are unambiguously demonstrated. Our system provides an ideal platform to explore the intriguing properties of acoustic waves endowed with extraordinary dispersions.

## Abstract (web summary)

3D topological nodal lines that give rise to drumhead surface states could help study a range of exotic topological phenomena. Here, Deng et al. experimentally demonstrate 3D nodal ring dispersion and topological drumhead surface states for acoustic waves in a phononic crystal.

## Introduction

The experimental discovery of Weyl points in electronic systems^1,2^ and photonic/phononic crystals^3,4^ prompted a great deal of research interest in topological semimetals^5,6^. Three-dimensional (3D) topological semimetals are characterized by band touching that carries nontrivial topology either at zero-dimensional discrete points or along one-dimensional continuum curves. Typically, point touching has different types such as Weyl point^7–9^, Dirac point^10^, and triple-point^9^, while curve touching, known as topological nodal line semimetals^11^, has a variety of geometrical configurations, such as nodal rings^12,13^, nodal chains^14^, and nodal links^15,16^. The nodal lines carry a nontrivial *π* Berry flux and produce drumhead surface states, which present numerous exotic topological transport properties^6^, such as scattering interference^17^ and resonant reflection^18^. Significant efforts, both theoretical^11–16^ and experimental^19,20^, have been made to realize nodal lines in condensed matter physics. However, these efforts remain challenging since the nodal lines are always sensitive to spin-orbit coupling and are easily merged into trivial bulk bands^21,22^. In particular, solid evidence for flat drumhead surface dispersion is still elusive because surface states are veiled into bulk bands in angle-resolved photoemission spectroscopy measurements^18,19^.

Motivated by band topology in condensed matter materials, the study of the topological properties of classical waves in artificial periodic structures is developing rapidly^3,4,22–33^. Gyroid photonic crystals are designed to realize Weyl points and nodal lines for electromagnetic waves^24,25^. Recently, the nodal chains/lines are observed in metallic-mesh photonic crystals/metacrystals^22,26^. However, the surface states in these systems show a large variation of frequency dispersion and substantially mix into the bulk bands. Thus, these systems cannot exhibit the drumhead profile as a whole. For acoustic waves, Weyl points have been theoretically predicted and recently observed in experiments^4,27^. Actually, acoustic waves are suitable for generating nodal lines due to the absence of intrinsic spin and spin-orbit interactions. An artificial phononic crystal (PC) can be an ideal platform for nodal line physics. To date, an acoustic nodal line has not been reported.

In this study, we propose a practical PC in three dimensions to measure the nodal rings and drumhead surface states predicted by a simple tight-binding model. The PC can be flexibly designed and accurately fabricated due to its macroscopic scale. The mirror symmetry of the PC protects the ring-like linear degeneracy. By directly measuring full pressure field distributions in the bulk and surface of the PC, nodal rings and drumhead surface states are evidently observed in reciprocal space. Numerical simulations and experimental observations consistently verify the existence of a phononic nodal ring material.

## Results

### Tight-binding model

**Fig. 1:**
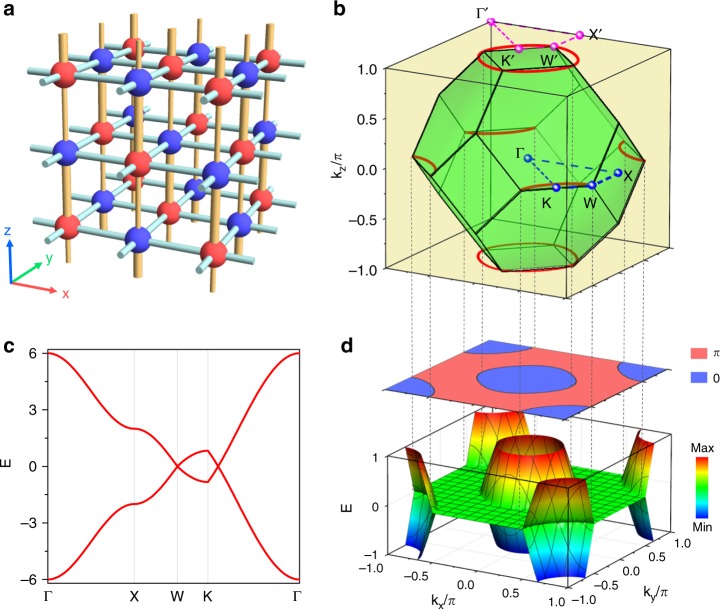
Nodal rings and zero modes for an fcc lattice model. **a** Schematic of the tight-binding configuration for the fcc structure. Red and blue spheres indicate the inequivalent lattice sites, which share uniform hoppings in the *x*–*y* plain (blue rods) and possess unequal hoppings along the *z* direction (yellow rods). **b** Nodal rings (denoted by red curves) distributed in momentum space. The green truncated octahedron corresponds to the first BZ in reciprocal space, where high symmetry points are marked. The high symmetry points with and without primes are differed by a reciprocal lattice vector. **c** Bulk dispersions in the *k*_*z*_ = 0 plane. Two dispersions linearly cross when passing the nodal rings, which also occur along an equivalent path in the *k*_*z*_ = *π* plane. **d** Upper panel: Zak phase distributions for the model. Lower panel: the associated drumhead surface dispersion at the boundaries in the *z* direction. The parameters for the model are *t* = 1 and *δt* = 0.4

A tight-binding model based on a face-centered-cubic (fcc) lattice is provided to illustrate the nodal rings and flat drumhead surface states. The lattice can be considered an array of dimer chains; each chain is an analogy of a typical Su–Schrieffer–Heeger (SSH) model. The hopping amplitude along the *z* direction is $- t \pm \delta t$ (*t* > 0 and *δt* > 0), which is represented by the smaller/larger yellow rod in Fig. 1a. The Hamiltonian of this model in momentum space is characterized by $H = d_x\sigma _x + d_y\sigma _y$, where $d_x = - 2{\mathrm{cos}}k_x - 2{\mathrm{cos}}k_y - 2t{\mathrm{cos}}k_z$, $d_y = - 2\delta t{\mathrm{sin}}k_z$ and σ_*x,y*_ are Pauli matrices that represent sublattice pseudospins. The nearest-neighbor hoppings (green rods) between distinct sublattices in the *x*–*y* plane are set to −1, and all distances between sublattices are set to unity. The eigenvalues of the Hamiltonian $E_ \pm = \sqrt {d_x^2 + d_y^2}$. When *d*_*x*_ = *d*_*y*_ = 0, band crossings occur and the crossing points form nodal rings in the extended Brillouin zone (BZ), as shown in Fig. 1b. The nodal ring that lies on the *k*_*z*_ = 0 (or *π*) plane obeys the contour ${\mathrm{cos}}k_x + {\mathrm{cos}}k_y = \mp t$. From a symmetry consideration, the existence of nodal rings is protected by the mirror symmetry on the *k*_*z*_ = 0 (or *π*) plane. The ring states possess opposite eigenvalues with respect to the mirror operation and avoid band repulsion^21^, which contributes to the linear crossings for the band dispersion, as shown in Fig. 1c. By considering *k*_*x*_ and *k*_*y*_ as parameters, the Zak phase of the model along the *k*_*z*_ direction is obtained as ${\mathrm{\gamma }} = ({\mathrm{\pi }}/2)\left[ {1 + {\mathrm{sgn}}\left( {t - \left| {{\mathrm{cos}}k_x + {\mathrm{cos}}k_y} \right|} \right)} \right]$. The Zak phase is *π* within the area (red region in upper panel of Fig. 1d) between the projections of the nodal rings on the *k*_*x*_ − *k*_*y*_ plane, which causes the existence of topological surface states. In the lower panel of Fig. 1d, flat drumhead surface dispersions are obtained in the *π* Zak phase region with rigid boundaries in the *z* direction. No topological surface states are observed in the *x* (or *y*) direction for the trivial Zak phase along the *k*_*x*_ (or *k*_*y*_) direction. The drumhead surface dispersions projecting to an arbitrary direction are discussed in Supplementary Note 1.

### Observation of nodal rings in a PC

**Fig. 2:**
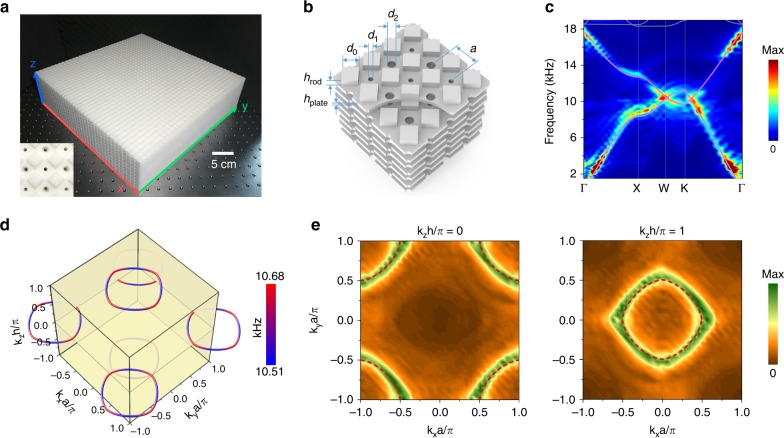
Nodal rings for sound waves in 3D phononic crystals. **a** Photograph of the 3D sample, with an enlarged front-view shown in the inset. **b** Schematics of a magnified side view of the sample, which is constructed by layered stacking structures. The thickness of the plate *h*_plate_ = 1.80mm in which staggered holes with diameters *d*_1_ = 2.16mm and *d*_2_ = 3.84 mm are penetrated and among which square pillars of height *h*_rod_ = 3.36mm and side length *d*_0_ = 7.20mm are periodically arranged. The distance between the staggered holes is *a* = 12mm, and the total height of a single layer *h* = *h*_plate_ + *h*_rod_. **c** The simulated (purple lines) and measured (colors) first and second bands along the high symmetry lines in the first BZ. Linear crosses occur at a frequency of approximately 10.6 kHz. **d** Simulated nodal rings distributed in reciprocal space. The colors denote the frequency variations, which range from 10.51 to 10.68 kHz. **e** Experimentally measured nodal rings at a frequency of 10.6 kHz shown at the planes *k*_*z*_*h*/*π* = 0 and *k*_*z*_*h*/*π* = 1, which agree with the simulated data (marked by red dashed curves)

The nodal rings are observed for acoustic waves in a 3D layer-stacked PC. The photograph of the sample is shown in Fig. 2a. The PC is layer-by-layer assembled with each layer fabricated by 3D printing. Sound waves can freely propagate in the void space of the structures. As shown by the enlarged schematic in Fig. 2b, the holes with different diameters, which contribute to unequal interlayer couplings, are alternately located on one plate. Each layer is supported by regular square pillars. The two diameters of the holes alternately change in the *z* direction. This structure for the interior acoustic waves can be modeled as the previously mentioned fcc lattice with staggered *z*-direction interactions.

The *D*_*4h*_ symmetry is retained for our PC and provides a mirror symmetry to guarantee the phononic nodal rings. To confirm the existence of the nodal rings, we measure the band structures in the first BZ by performing a Fourier transformation for the scanned acoustic pressure fields. Figure 2c shows the agreement between the simulated dispersions (purple lines) and measured dispersions (color maps) for the lowest two acoustic modes. The color maps represent the experimental dispersions expressed in terms of the average intensity of the Bloch states (Supplementary Note 2). The dispersions of the first two bands linearly cross at a frequency of approximately 10.6 kHz. An angle-resolved transmission measurement is further performed to manifest this feature, as given in Supplementary Note 3. A global view of the simulated nodal rings in reciprocal space is shown in Fig. 2d. Two nodal rings, at the planes *k*_*z*_*h* = 0 and *k*_*z*_*h* = *π,* respectively, are observed within the cubic reciprocal space. Note that the two rings are related by a translation of the reciprocal lattice, thus are the same. The nodal rings possess a frequency variation (only 0.6%, denoted by the color distribution on the rings) of approximately 10.6 kHz and yield satisfactory agreement with the tight-binding prediction. This slight frequency variation is intrinsic, thus irrelevant to the fabrication tolerance. Figure 2e shows the measured nodal rings with frequency 10.6 kHz. The experimental results (denoted by the colored data) coincide with the numerical simulations (red dashed curves) and are consistent with the theoretical predictions.

### Observation of phononic drumhead surface states

**Fig. 3:**
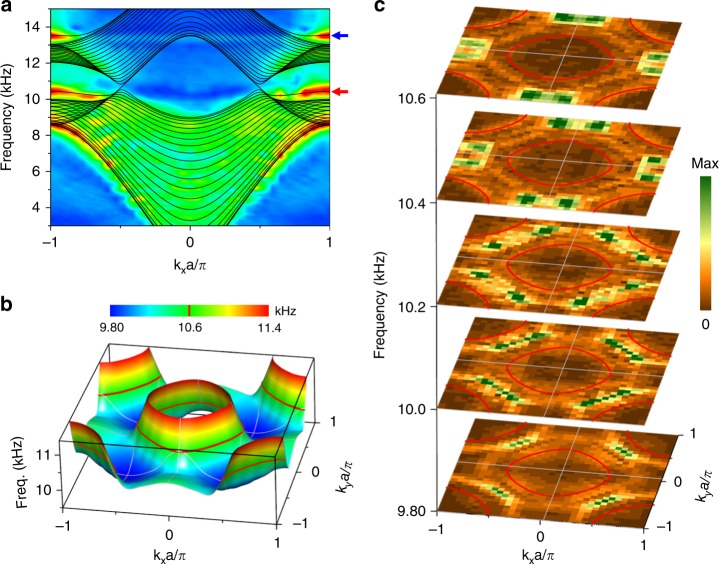
Phononic drumhead surface states. **a** The projected dispersion for *k*_*y*_*a* = 0 showing the surface states of the PC. The red arrows and blue arrows denote the drumhead surface states and high-frequency surface states, respectively. **b** A 3D view of the simulated dispersion for the drumhead surface states. The red lines show the equifrequency contour at 10.6 kHz. **c** Cross sections for the measured drumhead dispersion. The measured results are consistent with the variations of the simulated dispersion

The nontrivial topology of the nodal lines gives rise to drumhead surface states. The simulated projected surface dispersions are represented by black lines in Fig. 3a, where dispersions of the drumhead surface states occur at a frequency near 10.4 kHz. This finding is consistent with the observation that a drumhead exists between two projections of linear crossings of the nodal lines. Another surface state dispersion emerges at high frequency, near 13.4 kHz, and does not traverse the lowest two bands. Excited with a headphone at the center of the *x–y* surface of our sample, the dispersion of the surface states, including that of the projected bulk states, is observed by measuring and Fourier transforming the acoustic pressure distribution in the PC. The measured results coincide with the simulation results for both the drumhead surface state and the high-frequency surface state. We directly show the profile of the drumhead in *k*_*x*_ − *k*_*y*_ momentum space. In Fig. 3b, the drumhead dispersions, which are obtained by full wave simulation, are present between nodal ring frequency contours in two-dimensional reciprocal space. The drumhead dispersions are always beneath the nodal ring frequency (those shown above the nodal ring frequency are that of the bulk state) and exhibit a slight frequency variation (only 1.8%) due to the boundary potential induced by the practical structure (refer to Supplementary Fig. 5). The experimental measurements of the drumhead dispersions show great consistency with the numerical results, even the small frequency variations are adequately captured, as seen in Fig. 3c. As shown by the contour profiles, which range in frequency from 9.8 to 10.6 kHz, the drumhead dispersion in the ΓK direction with the lowest frequency is measured, the stimulated drumhead is slowly diffused to areas with higher frequency, and the drumhead near the X point is observed at the nodal ring frequency.

**Fig. 4:**
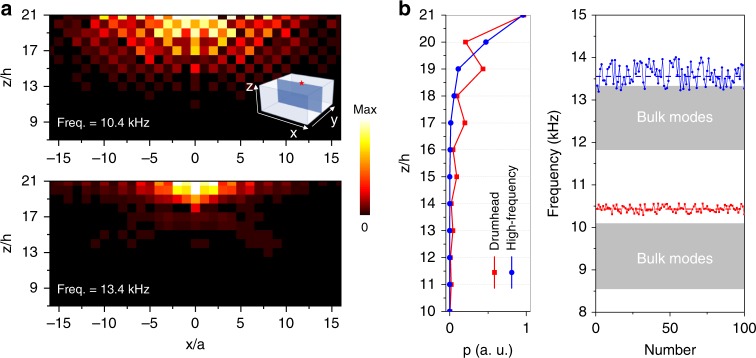
Exotic properties for the topological nontrivial drumhead surface states. **a** Acoustic pressure distributions in the depth direction experimentally scanned in a cross-sectional plane (denoted by the blue region in the inset). The red star marks the excitation point source. The fields at frequencies of 10.4 and 13.4 kHz exhibit distinctly different patterns for the drumhead surface state and the high-frequency surface state. **b** Left panels: the layer-average pressure fields for the surface states simulated in a square supercell. The drumhead surface state shows oscillated decay in contrast to the high-frequency state. Right panel: the frequency response in the presence of disorder for the supercell at (*k*_*x*_,*k*_*y*_) = (*π*/*a*,0). The drumhead surface state is more stable than the high-frequency state due to the topological protection

We distinguish the characteristics of the drumhead surface state from the characteristics of the high-frequency state. The measured drumhead acoustic field (upper panel, Fig. 4a) exhibits a stagger pattern in the depth direction, i.e., field strength primarily resides in one sublattice. This specific distribution stems from the nontrivial *π* Zak phase, which is akin to the end state in the SSH model. Conversely, high-frequency surface states (lower panel, Fig. 4a) reside along the *z* direction in both sublattices. These surface states are considered Tamm-like states^31^, which are produced by the effective defects caused by the boundary potential on the surface (see Supplementary Note 5). The different distributions for the drumhead and high-frequency states are explicitly demonstrated in the simulation of the layer-average pressure fields (left panel, Fig. 4a). Both surface states exponentially decay along the depth direction, whereas the drumhead state shows a damped oscillation. In the presence of weak disorder, where the disorder is added to the diameters of the staggered connecting holes as *d*_1,2_*w*_*r*_ with the random variable *w*_*r*_ uniformly distributed in the range from −0.15 to 0.15, the drumhead states (right panel, Fig. 4b) show only a slight variation because of the topological protection, whereas the topological trivial high-frequency states are unstable and spread into the bulk states.

## Discussion

We have directly observed nearly ideal nodal rings for acoustic waves in a 3D PC. A phononic drumhead surface state emerged in-between the nodal rings. Both the theoretical and experimental results of the nodal ring and the drumhead dispersions are in well agreement. Our work contributes to a 3D topological acoustic analog of the nodal line semimetal in addition to the recently discovered Weyl semimetal^4^. Benefit from the dense, very slow, and localized drumhead surface modes induced by the nodal rings, this artificial PC may give birth to innovative applications in acoustics, such as high-performance sound absorption and sensing on surface. Although we focus on acoustic realization in a specific layer-stacked PC, this design can extend to other types of classical waves and mechanical systems^32,33^.

## Methods

### Numerical simulations

All simulations were performed using the commercial COMSOL Multiphysics solver package. The systems are filled with air (with a density of 1.18 kg/m^3^ and speed of sound of 346 m/s at room temperature). The plastic stereolithography can be considered a hard boundary during the simulations due to the substantial acoustic impedance contrast compared with air. The bulk dispersions in Fig. 2 are obtained using one unit cell with periodic boundary conditions in all three directions. The surface dispersions in Fig. 3 are calculated using ribbon structures, where the rigid boundaries are imposed in the *z* direction, and the periodic boundaries are imposed in the *x* and *y* directions.

### Experimental measurement

The PC is prepared by 3D printing of plastic stereolithography material, which possesses dimensions of 30 × 430 × 115 mm and contains more than 12,000 periodic units. The PC is stacked 21 layers along the *z* direction, with each layer composed of designed structures that contain 35 × 35 unit cells in the *x*–*y* plane. The structures can be viewed as rigid walls for the acoustic wave that propagates in the PC due to the large impedance mismatch between air and the material. The measurement is performed by exciting the acoustic modes of the PC with an external loudspeaker and scanning the full acoustic pressure distributions in/on the bulk/surface of the PC. For acoustic excitation, a subwavelength headphone with a diameter of 6 mm addresses a frequency range that reaches 20 kHz. The headphone is placed at the center of the bulk sample for bulk state excitation, while the headphone is placed at the center of the *x–y* surface with a rigid boundary for surface-state excitation. To measure the acoustic fields, a microphone probe with a diameter of 1 mm is attached to the tip of a stainless-steel rod and inserted into the sample through the space between the plates and the scattering rods. The probe is controlled by a stage that moves in three directions. Sound signals (S-parameter S21) are sent and recorded by a network analyzer (Keysight 5061B). The bulk dispersions are obtained by Fourier transforming the scanned acoustic pressure field distribution inside the sample and constructing the excited Bloch states, as detailed in Supplementary Note 2.

## Supplementary information

Supplementary Information

## Data Availability

The data that support the findings of this study are available from the corresponding author upon request.
