# Supplementary material for: Nodal rings and drumhead surface states in phononic crystals
Source: Nat Commun. 2019 Apr 16;10:1769. doi: 10.1038/s41467-019-09820-8 (PMC6467985; doi:10.1038/s41467-019-09820-8)
Supplement: Supplementary file 1 — Supplementary Information [file 41467_2019_9820_MOESM1_ESM.pdf]

Supplementary Information for

"Nodal rings and drumhead surface states in phononic crystals"

Weiyin Deng, Jiuyang Lu, Feng Li, Xueqin Huang, Mou Yan, Jiahong Ma, Zhengyou Liu

Supplementary Figures 1 to 6
Supplementary Notes 1 to 5
Supplementary References

Supplementary Figures

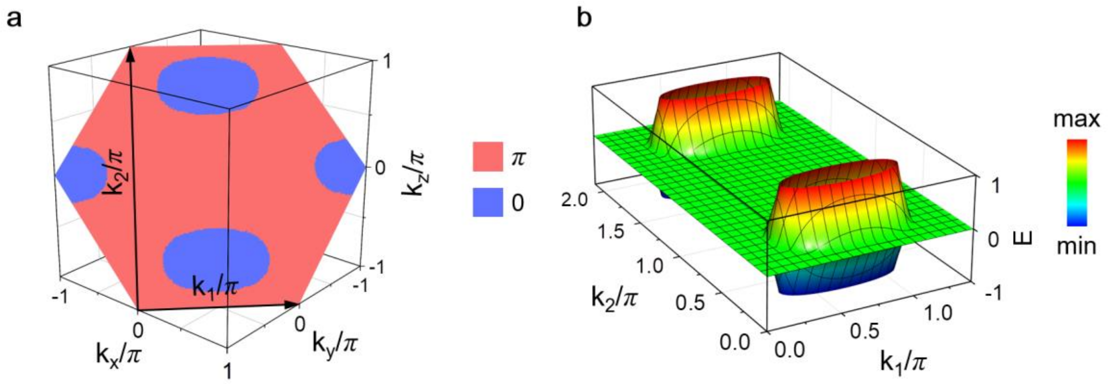

Supplementary Figure 1 | a, Zak phase distribution on the (111) plane calculated along [111] direction. **b**, Drumhead surface state dispersions on (111) surface, where colour scale represents energy eigenvalue in tight-binding model. Drumhead surface states exist in the region with nontrivial Zak phase, π.

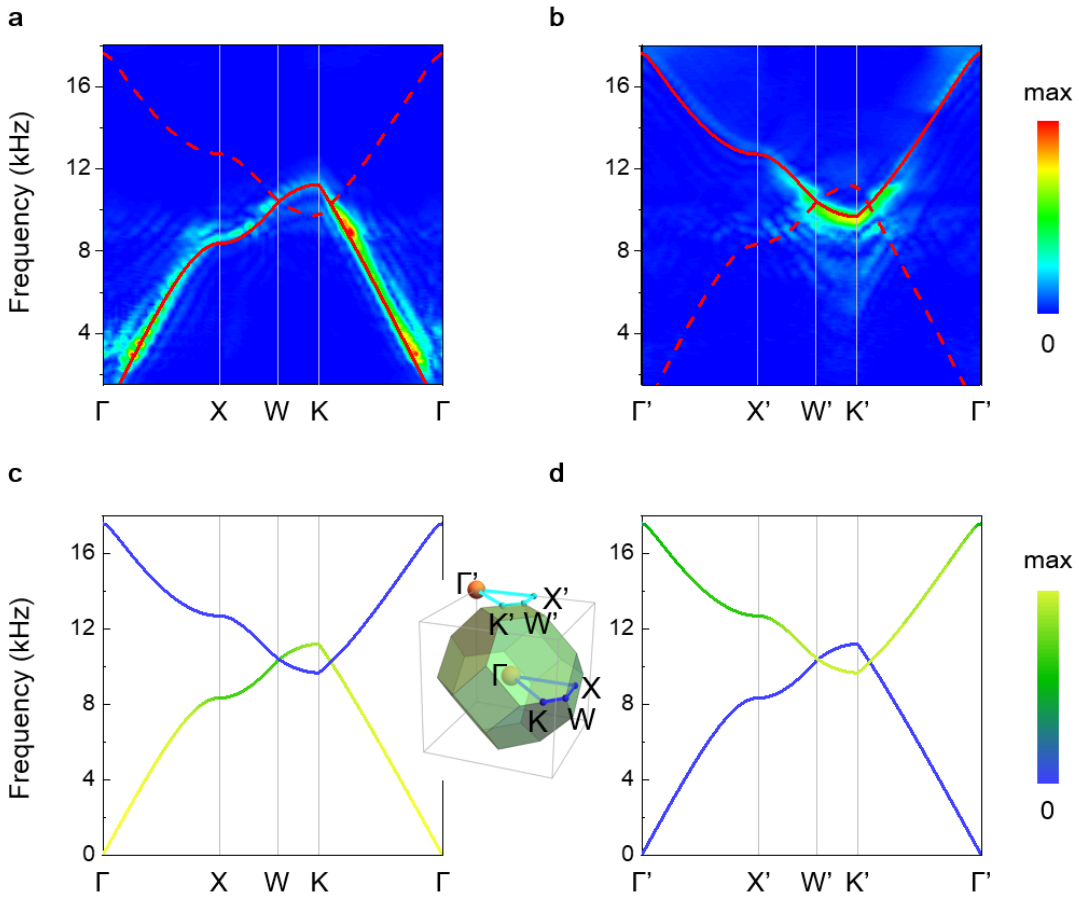

Supplementary Figure 2 | a and **b**, The simulated and measured bulk dispersions for the first two bands. **c** and **d**, The magnitude of projection from Bloch eigenstate of dispersions to plane wave state. Inset: the corresponding reciprocal space with high symmetry points marked. Here, the first band is visible in the first BZ and the second band in the second BZ is common, according to the band folding picture of the band theory.

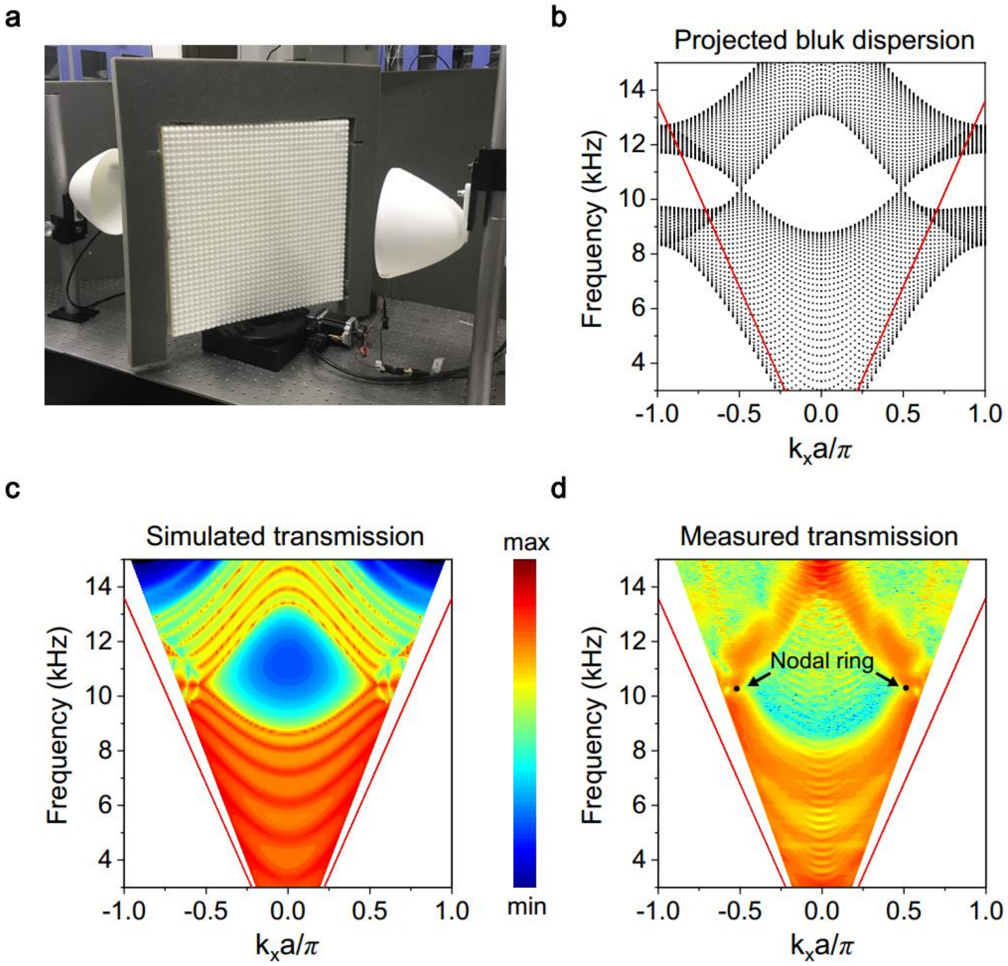

Supplementary Figure 3 | Angle-resolved transmission measurement of nodal ring bulk modes. a, The photography of the experiment setup, where a Gaussian acoustic wave is generated by the left speaker and the transmission is detected by the right one. **b**, The simulated dispersion projected along the k_x direction, where red lines delineate the sound cone. **c** and **d**, The simulated and measured transmissions. The first and second bulk bands touch at the crossing points near frequency 10.6 kHz.

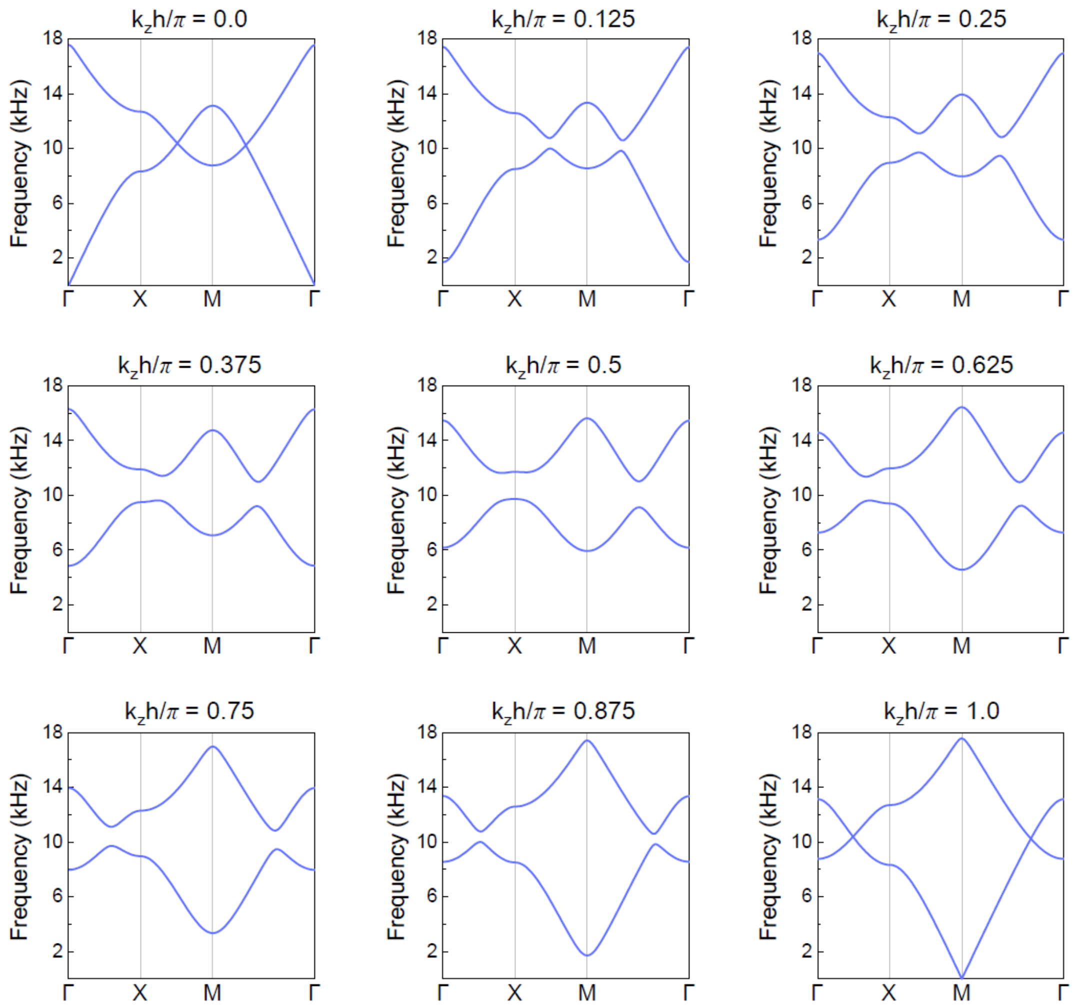

Supplementary Figure 4 | The simulated dispersions of the nodal line phononic crystal along high symmetry lines with different k_z, where $\Gamma = (0,0,k_z)$, $X = (\pi/a,0,k_z)$, and $M = (\pi/a,\pi/a,k_z)$.

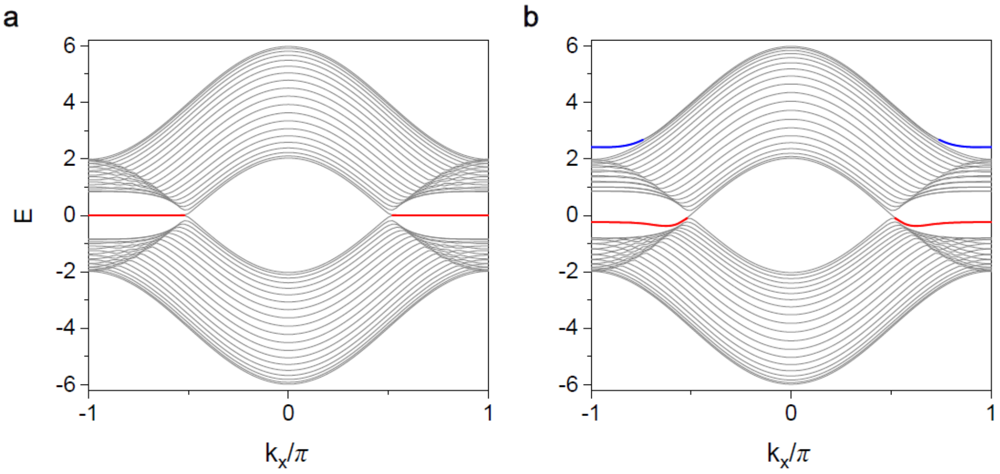

Supplementary Figure 5 | The theoretical surface states. a, The surface dispersion calculated using the standard tight-binding model with $k_y = 0$, where all the on-site energies are set zeros. **b**, The surface dispersion similar to **a** but with the correction of the on-site energy. The on-site energies of the first and second layer are corrected as $\varepsilon_{1A/1B} = -0.3/1.7$, $\varepsilon_{2A} = -0.6$, and the on-site energies near the other surface are same to this one in the *z* direction. The parameters are chosen as $t = 1$ are $\delta t = 0.4$. The size of the supercell is taken to be 20 layers with $N = 40$ sites.

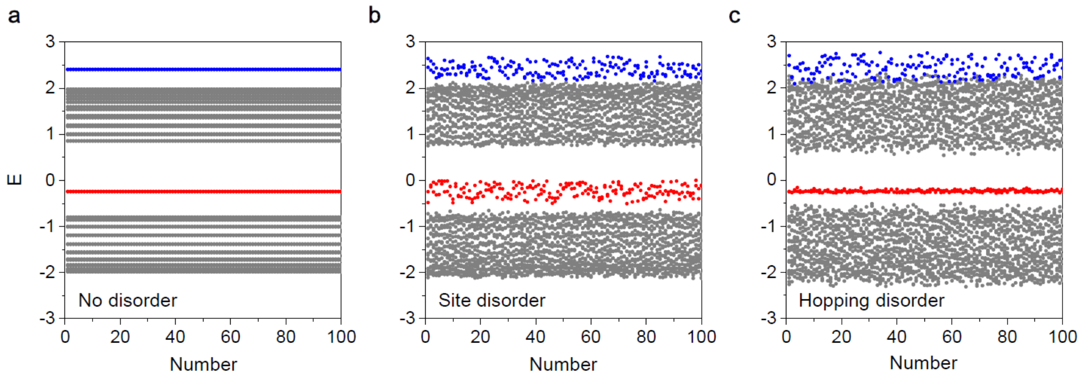

Supplementary Figure 6 | Eigenvalues in the presence of disorder. a, Band at $k_x = \pi$ and $k_y = 0$ in the absence of disorder, where the drumhead surface states are in the band gap and the high-frequency surface states are at the top of the band. **b**, Band at the same point with site disorder, where both the drumhead and high-frequency surface states are unstable and strongly fluctuated. **c**, Similar to **b** but with hopping disorder, where the high-frequency states are still unstable and turn to the bulk states, but the drumhead states are robust. Both of site and hopping disorder strengths are set to be $w = 0.6$. The other parameters are the same to Supplementary Figure 5b.

Supplementary Notes

Supplementary Note 1. Drumhead surface dispersions projecting to the $[\boldsymbol{l}, \boldsymbol{m}, \boldsymbol{n}]$ directions

In this section, we discuss the topological properties and drumhead surface dispersions projecting to an arbitrary direction. As shown in the text, for our design, the nodal ring lies on a horizontal plane. It projects to surface (l, m, n) as a deformed ring, as long as n is nonzero. Along a path in $[l, m, n]$ direction, if it hits the inside of the ring, the Zak phase is zero or trivial, otherwise it is π or nontrivial. Therefore, we can always expect the drumhead surface states in the area outside the ring on surface (l, m, n) with $n \neq 0$, such as (001) or (111). The Zak phase distribution and associated drumhead surface dispersion on surface (111) are shown in Supplementary Figure 1. However, for surface (l, m, n) with $n = 0$, such as (100), (010) or (110), the nodal ring projects to a line segment, the Zak phase along any path in $[l, m, n]$ direction, hitting the line segment or not, is uniformly zero or trivial, therefore, no drumhead surface states can be expected on these surfaces.

Supplementary Note 2. Experimental dispersion expressed in terms of average intensity of Bloch states

In this section, we provide a scheme to present the experimental dispersion in the first Brillouin zone (BZ), based on the Fourier spectra data obtained from experiment, such as Figs. 2c and 2e.

In experiment, the field or Bloch states is excited by a point source put inside the sample. The field distribution is obtained by scanning a tiny detector point by point inside the sample. Fourier transforming the field distribution gives the amplitude of all plane wave components (identified with $\boldsymbol{k}$) in the first BZ and in the extended BZs. From the spectra for all components, pick up the spectra for the components along path one (where $k_z = 0$) and path two (where $k_z = \pi/h$). Taking intensity of the spectra and plotting it in terms of path one and path two respectively, give the color maps in Supplementary Figures 2a and 2b.

It can be seen that, the band structure is well captured by the maxima of the spectra: the first band is captured by the maxima in the first BZ, as seen Supplementary Figure 2a, while the second band is captured by the maxima in the second BZ, as seen in Supplementary Figure 2b. The experimentally captured bands in the two BZs are denoted by red solid lines, while those not captured by experiment in the two BZs are denoted by red dashed lines. However, the fact that the second band is not captured by the experimental spectra (Supplementary Figure 2a) in the first BZ doesn't mean that the Bloch states in this band cannot be excited, and it just means that the Bloch states in the second band have vanishing plane wave component in the first BZ (Supplementary Figure 2c). Similarly, that the first band is not captured by the experimental spectra in the second BZ (Supplementary Figure 2b) doesn't mean that the Bloch states in the band cannot be excited, and it just means that the Bloch states in the first band have vanishing plane wave component in the second BZs (Supplementary Figure 2d). Actually, the Bloch states in both bands are excited in the experiment, as revealed by the maxima in the first BZ (the first band), and the maxima in the second BZ (the second band). In other words, the Bloch states in the first band are dominated by the plane wave component in the first BZ, while the Bloch states in the second band are dominated by the plane wave components in the nearest second BZs.

As the Fourier spectra correspond to the plane wave components of the excited field or Bloch states, not to the excited Bloch states themselves, clearly, the Fourier spectra in the first BZ only, are in general not enough to give the full band structure,

unless the Bloch states in all bands have observable plane wave components in the first BZ, which is impossible according to the band theory. Although the experimental Fourier spectra do not directly give the Bloch states but their components, however we can construct the Bloch states in each band by picking up and assembling the corresponding plane wave components in all BZs from the complete Fourier spectral data. With the attainment of the Bloch states, the band structure can be expressed in terms of Bloch states themselves rather than their components, and what we only need to do is to calculate and plot the average intensity in the first BZ for all Bloch states. In this way, experimental band structure has successfully been expressed in a single BZ, as Fig. 2c in the main text.

In addition, the mirror symmetry influences the visibility of some bands. Specifically, for the second band in our case, since the Bloch modes are antisymmetric with respect to the horizontal mirror plane, the Bloch modes along Γ-X-W-K-Γ must have vanishing plane wave components in the first BZ, because those plane wave components are symmetric with respect to the mirror plane, violating the symmetry of the Bloch modes. This account for the non-visibility of the second band in the first BZ.

Supplementary Note 3. Angle-resolved transmission measurement of nodal ring bulk modes

In this section, an angle-resolved transmission measurement[1] is performed to show the projection of the nodal line bulk modes. The set-up of the experiment is show in Supplementary Figure 3a. The phononic crystal is placed on a rotatable platform. A Gaussian wave generated by a parabolic speaker incident into the phononic crystal from the left side, and the transmission signal, as a function of incident angle, is detected by an identical speaker on the other side. In general, the transmission spectrum should capture the projected bulk dispersion within the sound cone lines as delineated by the red lines in Supplementary Figure 3b. The simulated and measured transmission spectra with respect to the in-plane wavevector k_x are shown in Supplementary Figures 3c and 3d. Near the frequency of 10.6 kHz, the crossing points between the projections of the first and second bulk bands are exactly the projections of the nodal rings with $k_y = 0$. Note that, compared to the projected bulk dispersion showing clear crossings, both the simulations and measurements show non-vanishing transmissions extended from the crossing points to the gaps on the left and right, which is due to the excitation of the surface modes on the front and back surfaces. But in any case, band crossing is clear, and the simulation and measurement show good agreement.

Supplementary Note 4. The simulated bulk dispersions of the nodal ring phononic crystal

In this section, we show the simulated dispersions along a high symmetry path to show the semimetal nature of the phononic crystal, where the bands are touched only at the ring nodes and gapped for the other Bloch wavevectors. The dispersions for different k_z are shown in Supplementary Figure 4, where the high symmetry paths are indicated by the points $\Gamma = (0,0,k_z)$, $\mathrm{X} = (\pi/a, 0, k_z)$, and $\mathrm{M} = (\pi/a, \pi/a, k_z)$. It can be seen that, the nodal ring initially surrounds the $\mathrm{M} = (\pi/a, \pi/a, 0)$ point, and as k_z increases, the ring changes to the surround the $\Gamma = (0,0,\pi/h)$ point. All in this process, the gap between the first and second bands remains open, leading a significant feature of semimetal phononic crystal. These results demonstrate clearly that nodal ring degeneracy between the lowest two bands only appears at $k_z = 0$ and π/h.

Supplementary Note 5. The origin of the high-frequency surface state

In this section, we discuss the high-frequency surface state exits in our 3D PC structure. The high-frequency surface is similar to the new type of edge state in photonic graphene lattices[2], which is at the top of the band and dose not cross with the other bulk projected dispersions. The high-frequency surface dose not exit in Supplementary Figure 5a, which is calculated directly from the model described by the tight-binding Hamiltonian in the main text. However, when effective defects are considered on the lattice surface, the high-frequency surface state appears as the blue line denoted in Supplementary Figure 5b, which emulates the simulation and experimental results in Fig. 3a of the main text. In addition, the effective defects also make the drumhead dispersions (red line) slightly distorted. As explained in ref 2, the effective defects can be attributed to the correction of the on-site energy near the surface, because of the boundary potential induced by the practical structure. The sites near the surface are significantly different from the bulk sites in a finite lattice, giving rise to the effective potential on the lattice surface. Therefore, this surface state is classified as a Tamm-like surface state, since Tamm states are associated with surface defects or perturbations.

In order to demonstrate the essential difference between the drumhead surface state and the high-frequency surface state, we study the disorder effect in the tight-binding model. The disorder is only considered in the *z* direction with open boundaries, and k_x and k_y are still good quantum numbers for the lattice periodicity in the *x*-*y* plane. Given $k_x = \pi$ and $k_y = 0$, we plot the dispersion in the absence of disorder in Supplementary Figure 6a. One can see that the drumhead surface states are in the band gap, while the high-frequency surface states are at the top of the band, which corresponds to band at $k_x = \pi$ in Supplementary Figure 5b. We introduce site energy disorder as $\varepsilon_{\text{rand}} = w\,\xi/2$ and hopping integral disorder as $t^{\pm}_{\text{rand}} = t_{\pm} w\,\xi/2$, respectively, where $w = 0.6$ is the disorder strength, ξ is a random number uniformly distributed in -1 to 1, and $t_{\pm} = -t \pm \delta t$ are the staggered hopping integrals along the *z* direction. In Supplementary Figure 6b, we show the calculation results with 100 disorder configurations in the present of site energy disorder. Both the drumhead surface states and high-frequency surface states are unstable. The site disorder generates a term like $d_z\sigma_z$ in the Hamiltonian, which breaks the chiral symmetry and makes the Zak phase ill-defined. As a result, the system is no longer protected by the topological invariants and thus unstable against such disorder. In

contrast when we introduce the hopping integral disorder, the drumhead surface states are robust against disorder, whereas the high-frequency surface states are still unstable, as shown in Supplementary Figure 6c. The hopping disorder only slightly changes the $d_x\sigma_x$ and $d_y\sigma_y$ terms, and does not cause a topological transition, so that the drumhead surface state can survive. Therefore, the drumhead surface states are topologically protected, and the high-frequency surface states are topologically trivial.

Supplementary References

1. Q. Yan, R. Liu, Z. Yan, B. Liu, H. Chen, Z. Wang, L. Lu, Experimental discovery of nodal chains. *Nat. Phys.* **14**, 461-464 (2018).
2. Y. Plotnik, M. C. Rechtsman, D. Song, M. Heinrich, J. M. Zeuner, S. Nolte, Y. Lumer, N. Malkova, J. Xu, A. Szameit, Z. Chen, M. Segev, Observation of unconventional edge states in 'photonic graphene'. *Nat. Mater.* **13**, 57-62 (2014).
